# The COVID-19 pandemic and its impact on health experiences of asylum seekers to the United States

**DOI:** 10.1186/s12889-023-16313-3

**Published:** 2023-07-18

**Authors:** Elizabeth Singer, Kevin Molyneux, Mahalya Gogerly-Moragoda, Dustin Kee, Kim A. Baranowski

**Affiliations:** 1 Departments of Emergency Medicine and Medical Education, Icahn School of Medicine at Mount Sinai, 1111 Amsterdam Avenue, New York, NY 10025 USA; 2 Department of Emergency Medicine, Columbia University, New York, USA; 3 Icahn School of Medicine at Mount Sinai, New York, USA; 4 Department of Medical Education, Icahn School of Medicine at Mount Sinai, New York, USA

**Keywords:** COVID-19, Asylum seekers, Healthcare, Immigration, Disparities

## Abstract

**Background:**

The COVID-19 pandemic exacerbated preexisting barriers to accessing healthcare and social services faced by asylum seekers to the United States. This study aimed to uncover the impact of the first year of the COVID-19 pandemic on asylum seekers, including socio-economic stressors and access to medical information, healthcare, and testing.

**Method:**

We conducted 15 semi-structured, in-depth interviews with adult asylum seekers to the U.S. and systematically analyzed the resulting transcripts using a consensual qualitative research approach.

**Results:**

The transcripts yielded six domains: (1) knowledge and understanding of COVID-19; (2) attitudes and practices relating to COVID-19 precautions; (3) experience of COVID-19 symptoms; (4) current physical and mental health; (5) access to and interaction with health care; (6) discrimination based on asylum status.

**Conclusions:**

Although participants had knowledge about COVID-19’s communicability and regularly used masks, their living conditions frequently hindered their ability to quarantine and isolate, and their lack of insurance was often a deterrent to them seeking medical care. Notably, immigration status was not a significant factor discouraging participants from seeking care during the pandemic. The findings build on existing knowledge about this community and may help define areas where support and services can be expanded in current and future pandemics.

## Introduction

The COVID-19 virus has dominated nearly every aspect of human life since emerging in 2019. The virus has been responsible for over 750 million cases and close to seven million deaths globally, as reported by the World Health Organization in 2023 [1]. It has disrupted supply chains, health care access, and caused extensive financial concerns across the globe [2]. Amid the myriad effects of the pandemic, migrant populations in particular have been underrepresented in research data, despite being heavily affected by the virus. Rates of COVID-19 transmission have, in fact, been significantly higher in refugee and asylee populations [3]. In addition to being at an increased risk for COVID-19 infection, migrant populations in high-income Western countries have a disproportionate mortality from COVID-19, which has been linked to high-risk occupations, overcrowded living accommodations, and obstacles to healthcare such as lack of information, language barriers, and fewer resources than the general population [4].

The death toll alone, however, does not capture the collateral effects on health equity, such as loss of jobs, insurance, housing, and lack of access to basic needs [5] (experienced by forced migrants. Refugee populations are 60% more likely to work in sectors highly impacted by COVID-19, with resultant disproportionate job losses; they also lack the same access to governmental aid and relief as non-refugee populations [6]. Those who do remain in their jobs are more likely to work in fields with higher COVID-19 exposures and are often unable to work remotely or stay home from work [7]. At home, asylum seekers experience increased exposure to COVID-19 due to living in overcrowded housing and shared accommodations, making it difficult to adopt preventative measures such as social distancing, quarantining, and isolating if ill [8]. Latinx patients who reported Spanish as their primary language, were younger, and had a larger household size experienced an increased rate of testing positive for COVID-19 [9]. Research has shown that those experiencing more taxing living situations may also encounter greater obstacles to following precautions designed to prevent the spread of COVID-19 [10].

Although there has been some quantifying data on the effect of the pandemic, migrant populations in the United States have traditionally been underrepresented in research. First, the health of asylum seekers and their interactions with healthcare, in particular, has been an under-studied field [11]. In addition, studies that do exist often fail to capture immigrant and refugee population data, and adjustments must be made in study designs to specifically engage these groups and establish a reliable sampling frame, often through such means as a government database [12]. Furthermore, the U.S. has a larger immigrant population than any other country in the world [13]. The most recent governmental statistics come from fiscal year 2019, the year before the pandemic, when a total of 29,916 people were newly admitted to the U.S. as refugees, and an additional 46,508 were granted asylum status from over 90,000 asylum applications [14].

Yet, what is known about immigrant populations is that they faced health disparities stemming from systemic forces since well before the pandemic, including lower rates of access to health insurance, lower health care utilization, and lower quality of care than U.S.-born populations [15]. U.S. policy decisions have resulted in restrictions on access to public health insurance programs, decreased caps on the number of admitted refugees, and fewer protections from deportation, contributing to a growing public health crisis in this population [16]. The Public Charge Rule, which went into effect under the Trump Administration specifically stated that “aliens were inadmissible to the U.S. if they had been unable to care for themselves without becoming public charges,” and had relied on government services. This led some parents to disenroll their children from programs such as Supplemental Nutrition Assistance Program (SNAP). Although this public charge policy does not apply to testing for COVID-19, prior experiences contributed to immigrant communities’ distrust of the government and impacted their receptiveness to public health messaging [17].

Amongst arriving refugees and asylum-seekers, there has also been a demonstrated discrepancy in health literacy around COVID-19. A study of asylum seekers at a temporary camp in Matamoros, Mexico indicated that about 50% of the participants noted importance of masking, social distancing, and hand hygiene, but had encountered misinformation about COVID-19 and the potential risks of the virus affecting them [18]. It further appears that there is little governmental guidance regarding asylum seekers at the U.S.-Mexico border and in-transit migrants within Mexico [19]. In the U.S., there has been a lack of culturally appropriate and linguistically competent information for immigrant communities, forcing patients to rely on social media for guidance which may contain erroneous health messaging [20]. Safety concerns may also have contributed to decreased effectiveness of public health messaging and a need to provide assurances to asylum seekers that access to vaccination does not include inquiries into immigration status and the threat of deportation [8].

As a result of these factors, the stresses of the pandemic are associated with far reaching mental health outcomes. COVID-19 has led to increased rates of clinically significant psychological distress in the general international population [21]. Even prior to the pandemic, refugee and asylee communities in Western countries were about ten times more likely to be diagnosed with post-traumatic stress disorder (PTSD) than age-matched general populations in those countries [22]. There are also substantial numbers of asylum seekers and refugees who experience significant psychological symptoms, but do not receive mental health diagnoses or services [23].

It is within this complex context that we sought to qualitatively investigate the effects the pandemic has had on refugee and asylee communities. We focused on capturing COVID-19’s impact on their health, access to health care, understanding of the pandemic, disruptions to daily life, and the ability to take preventative measures against COVID-19.

## Method

This consensual qualitative research (CQR) study was approved by the Icahn School of Medicine at Mount Sinai Institutional Review Board and eligible participants included asylum seekers over the age of 18 living in the U.S. during the COVID-19 pandemic. Recruitment emails in English, Spanish, and French were sent to legal, and human rights organizations that serve asylum seekers and with which the Mount Sinai Human Rights Program partners. Study participants were referred from fifteen such legal teams or human rights organizations located in New York, New Jersey, Connecticut, and Texas. These communications included a description of the research, inclusion criteria, and contact information for the principal investigator. Interested participants provided informed consent and responses to a brief demographic survey, before completing a one-hour semi-structured, audio-recorded telephone interview (Table 1) which focused on participants’ knowledge, attitudes, and health practices during the early stages of the COVID-19 pandemic, their access to health care, and their employment experiences and living conditions. The interviews were facilitated by either a physician or psychologist with experience working with asylees. The resulting transcripts were deidentified in order to maintain confidentiality and to protect personal health information before analysis.

**Table 1:** Semi-structured interview protocol

1. Please describe what you know about COVID-19 (i.e., transmission, symptoms, affected body parts/systems, high-risk groups)
2. Please describe what you know about the ways COVID-19 might be prevented
3. What can people do to protect against COVID-19? In what ways have you been able, or not been able, to protect yourself?
4. Please describe where you receive most health information about COVID-19
5. Since March 2020, please share whether you ever thought you had COVID-19/Coronavirus. If so, what was your experience (i.e., symptoms, access to testing and treatment, ability to isolate, outcome)?
6. Since March 2020, please share whether you ever thought someone in your immediate household had COVID-19/Coronavirus. If so, what occurred (i.e., symptoms, access to testing and treatment, ability to isolate, outcome)?
7. Since March 2020, what experiences of discrimination do you believe you encountered because you are an asylum seeker, if any
8. Is there anything else you would like us to know about your experience during the COVID-19 pandemic?
9. What was it like for you to participate in today’s interview?

Between December 2020 and February 2021, 15 asylees to the U.S. volunteered to participate in the study, the sample size fell within the recommended range of 13–15 individuals for a CQR analysis [24]. The research team adhered to the CQR process [24] in their analysis of the data, beginning with the development of domains that organized the participants’ narratives into general themes. Next, they summarized the transcript text into core ideas or brief summaries and compared participants’ responses while generating categories that organized these experiences in more detail. Then, they assigned frequency labels that described the representativeness of each category: “general” categories contained 14 to 15 participants, “typical” categories contained 8–13 participants, and “variant” categories contained 2–7 participants. “Rare” categories reflected the experiences of only one participant and are not reported as they are not considered to be representative of this study’s sample.

At each stage of the CQR process, the researchers worked to minimize bias and interpretation by first coding the data independently, before meeting to argue each code to consensus. Furthermore, an external auditor experienced in this method, reviewed the analysis and provided feedback and suggestions to increase the accuracy and trustworthiness of the coding. In addition, the team returned to the raw data and identified descriptive participant quotations to ensure that the analysis was grounded in the words of the asylum seekers interviewed.

## Results

The participants identified as cisgender women (*n* = 8, 53%) and cisgender men (*n* = 7, 47%). They ranged from 21 to 46 years of age (*M* = 36 *SD* = 7). They had lived in the U.S. less than one year (*n* = 2, 13%), one to three years (*n* = 6, 40%), three to five years (*n* = 5, 33%), or more than five years (*n* = 2, 13%). They noted that their asylum status was either still pending (*n* = 10, 67%) or granted (*n* = 5, 33%). They reported that they immigrated from nations in West Africa (*n* = 4, 27%), East Africa (*n* = 3, 20%), Central America (*n* = 3, 20%), the Caribbean (*n* = 2, 13%), Europe (*n* = 1, 7%), South America (*n* = 1, 7%), and North America (*n* = 1, 7%). Interpreters were utilized when appropriate, as the participants were interviewed in their preferred language: English (*n* = 9, 60%), Spanish (*n* = 4, 27%), and French (*n* = 2, 13%). They reported their highest education level as some primary school (*n* = 1, 7%), completed high school (*n* = 2, 13%), some college/vocational school (*n* = 1, 7%), completed college (*n* = 4, 27%), and completed graduate school (*n* = 6, 40%).

The analysis resulted in the creation of six domains representing the overarching themes contained in the asylum seekers’ narratives: a) experience of discrimination; b) current physical and mental health; c) knowledge and understanding of COVID-19; d) attitudes and practices to protect against COVID-19 transmission; e) experiences of COVID-19 symptoms; and f) access to and interaction with the health care system. These domains contained 19 categories (Table 2).

**Table 2:** Domain and category frequencies

Domains	Categories	Frequency Label
Experience of Discrimination		Typical (*n* = 11)
	Discrimination due to asylum seeker status	Typical (*n* = 8)
	Discrimination in employment	Variant (*n* = 6)
	Discrimination in housing	Variant (*n* = 3)
Current Physical and Mental Health		
	Increase in psychological distress	Typical (*n* = 9)
	Experience of chronic medical condition	Variant (*n* = 7)
Knowledge and Understanding of COVID-19		
	Received information through media	General (*n* = 15)
	Identified COVID-19 symptoms	General (*n* = 15)
	Symptoms develop within 14 days	General (*n* = 15)
	Virus is spread through close contact with others	General (*n* = 14)
	Social distancing can reduce transmission	General (*n* = 14)
	Good hygiene can reduce transmission	Typical *(n* = 13)
	Face masks can reduce transmission	Typical (*n* = 12)
	Quarantine can reduce transmission	Variant (*n* = 2)
Attitudes and Practices to Protect Against COVID-19 Transmission		
	Regularly wore face masks	General (*n* = 15)
	Living with others	General (*n* = 14)
	Confined living quarterly prevented social distancing	Variant (*n* = 7)
Experiences of COVID-19 Symptoms		Variant (*n* = 7)
Access to and Interaction with the Health Care System		
	Fear impacted access to care	Typical (*n* = 8)
	Access to health insurance	Variant (*n* = 7)
	Access to primary care physician	Variant (*n* = 6)

2–7 participants (variant); 8–13 participants (typical); 14–15 participants (general)

### Experience of discrimination

Most of the interviewees reported that they have been targeted with discrimination in the U.S. (*n* = 11), with many identifying experiences of oppression directly related to their status as an asylum seeker (*n* = 8). As one participant stated, “When they know we are asylum seekers, they treat us differently. Not in a good way.” (P6) In some cases, they noted that they experienced discrimination due to their language (*n* = 2), “I’ve been trying to speak with my poor English. This is what I noticed – that when they realize, they just change their attitude [toward me].” (P2) Another added, “I’m a lawyer. I’m a human rights activist. If I could express all the things in English that I know in Spanish, I could say a lot… people have the right to have dignity, to have help.” (P6) A few also described discrimination due to being forced to wear an ankle monitor by Immigration and Customs Enforcement officials (*n* = 2). One participant explained, “There’s nothing I could do [with this ankle monitor]… I feel like I’m not a normal person.” (P11) Another asylum seeker shared, “Whenever people see my ankle bracelet, they think I have a criminal record, so I cannot get work, not even one day, and it's horrible.” (P4).

The asylum seekers also reported they were targeted with discrimination in their places of employment (*n* = 6). One interviewee noted that during the early phase of the pandemic they faced specific aspects of work discrimination. “A lot of the rules about which restaurants are allowed to open, has led a lot of workers like myself who are working under the table, to be paid cash. It has allowed us to be exploited along the way. I work six days a week… from 9:00 am to 9:00 pm and I get paid $300 a week. I know that we are not here legally, we are not working legally, but we are here, and we are working.” (P14) Participants also reported experiencing housing discrimination (*n* = 3), as exemplified by one interviewee’s statement, “as an asylum seeker, the financial condition I am in, it is not good. I was trying to get some help in every way for housing, like any kind of support for the poor, but I didn’t get any help.” (P6) Another added, “because I didn’t have legal status, I wasn’t getting unemployment, I wasn’t getting the stimulus bill.” (P14).

### Current physical and mental health

Approximately half of the asylum seekers shared that they experienced chronic medical conditions (*n* = 7). Specifically, they reported high blood pressure (*n* = 4), high cholesterol (*n* = 2), HIV (*n* = 2), sleep apnea (*n* = 1), thalassemia (*n* = 1), and asthma (*n* = 1). However, as they reflected on their experiences during the interviews, they emphasized the increase in psychological distress they experienced as a result of the COVID-19 pandemic (*n* = 9).

They noted that they experienced exacerbation of their symptoms related to anxiety (*n* = 4), depression (*n* = 2), and posttraumatic stress disorder (*n* = 1) during the pandemic. One interviewee explained, “my level of anxiety was high, because I needed to protect myself more and I could infect others [with COVID].” (P5) In addition, they stated that they felt lonely (*n* = 2) and trapped (*n* = 1), as well as grief for loved ones who had died (*n* = 1). A participant disclosed, “Before the pandemic, I used to see a group of my friends every weekend. We would go out together and just talk, and laugh, and share ideas, and eat together. Since March, we haven’t been able to do that, so when we want to talk to our friends, we can call them or video call them if you want to see them with your eyes, but it’s still not the same. It’s been a strange world for me, one that I find very desolate and sad, and especially sad for people who have been affected or have lost their lives. It’s just very regrettable.” (P7) They also noted stressors associated with food insecurity increased during the pandemic, as exemplified by one participant’s statement, “It was food [that concerned me], especially food for my children.” (P9) Another added, “I didn’t have enough food or anything… it was a very dire situation for me.” (P8).

### Knowledge and understanding of COVID-19

All of the asylum seekers interviewed indicated that they received information about the COVID-19 pandemic through media (*n* = 15), specifically television (*n* = 10), the internet (*n* = 9), and social media platforms (*n* = 4). All of the participants in this study remarked that COVID-19 symptoms develop within 14 days following exposure (*n* = 15). Almost all of the interviewees reported that the virus is spread through close contact with others (*n* = 14). They noted that the lungs were most affected by the virus (*n* = 14) and that the elderly (*n* = 12), as well as those with pre-existing medical conditions (*n* = 12) were particularly vulnerable to experiencing severe illness. All of the asylum seekers identified a range of symptoms associated with COVID (*n* = 15), including fever (*n* = 12), muscle aches (*n* = 9), SOB (*n* = 6), loss of smell (*n* = 6), headache (*n* = 6), loss of taste (*n* = 5), flu-like symptoms (*n* = 4), diarrhea (*n* = 4), nasal congestion (*n* = 2), and chest pain (*n* = 2).

All of the interviewees stated that to avoid COVID-19 transmission, individuals should engage in social distancing (*n* = 14). They also reported that they should wear face masks (*n* = 12) to reduce the spread of the virus. Additionally, they stated that they believed that good hygiene (*n* = 13), specifically frequent hand washing (*n* = 12) and surface disinfection (*n* = 6), is essential to prevent infection. One participant summed it up as, “one has to wear a mask, also one has to socially distance, six feet. Maintaining efficient hygiene, cleaning, using disinfectant… wearing gloves and PPE.” (P3) Lastly, a few of the asylum seekers indicated that quarantine following exposure is needed to reduce the risk of spreading the virus (*n* = 2).

### Attitudes and practices to protect against COVID-19 transmission

Almost all of the asylum seekers in this study reported living with others (*n* = 14) with a majority sharing rooms (*n* = 12), including their sleeping areas (*n* = 7). As a result, almost half of the participants reported that their experiences of confined living quarters or reliance on public transportation prevented them from engaging in social distancing or isolating (*n* = 7). One asylum seeker reported, “I knew that [my sister] was sick. We were living just in one room; we didn’t have the space to isolate.” (P6) A second participant explained, “I would say the people that are vulnerable in the housing, they are in the most affected demographic, because they cannot isolate themselves from other people, or they don’t have private transportation like a car or anything, so they are in a horrible situation for COVID.” (P8) All of the interviewees reported that they regularly wore face masks to prevent COVID-19 exposure (*n* = 15). One asylum seeker asserted, “I feel good about wearing a mask.” (P15) Many also commented that they encountered challenges while wearing face coverings (*n* = 9). As one participant noted, “I feel inconvenience, but it’s probably protecting my life…. I end up having no choice to put it on to save my life and also to protect others, and to make sure others feel secure when I am next to them or around them.” (P12) Another interviewee described communication difficulties and reported, “I'm hard of hearing, so I always have a hard time with interrupting people wearing masks. So that's been a dilemma for me.” (P3).

### Experience of COVID-19 symptoms

Approximately half of the participants reported experiencing COVID-19 like symptoms (*n* = 7). One interviewee explained, “I had a sore throat and body aches, I was coughing, sneezing, I lost my taste when I was eating. I also lost my appetite.” (P12) Another stated, “I felt very feverish, my temperature was very high, and it was also difficult to breathe, difficult to sleep.” (P1) Many of the asylum seekers interviewed indicated that they had accessed a COVID-19 test during the pandemic (*n* = 11), but others noted that they experienced obstacles. As one participant reported, “I didn’t know where I could get the test. They told us we needed an appointment. We called the clinic, but the telephone rang and rang, and no one answered. Someone told me something about a website, but I couldn’t find it.” (P16) Another participant noted that they were evicted when they tested positive, “when my landlord knew that I had COVID, he didn’t allow me to stay there, so I had to go to the homeless shelter, and from the shelter, they sent me to the hotel program for isolation.” (P8) One asylum seeker added that, because they had been forcibly separated from their families when they fled their country of origin, they were fearful there would be no one to identify them if they died from COVID, “I was scared I thought that I was going to die and then my body goes John Doe, because I am in this country by myself.” (P12).

### Access to and interaction with health care system

The asylum seekers in this study reported a range of barriers to health care access during the pandemic. Less than half (*n* = 7) stated that they had health insurance at the start of the pandemic. A participant explained, “I wouldn’t have gone [for treatment], because I don’t have insurance.” (P7) Additionally, a smaller number reported that they had a primary care physician (*n* = 6), which led several to rely on emergency department visits (n = 4). Many noted that fear impacted their ability to access care (*n* = 8), specifically concerns related to increased risk of COVID exposure in medical settings and lack of effective treatment (*n* = 6). One interviewee disclosed, “​​I never thought about going to a hospital or a facility because I heard and I read they were so crowded.” (P2) Another participant explained, “I wanted to [go to the hospital], but then I heard… that people are being left to die” (P1) Others reported that worries related to that immigration status impacted their access to health care during the pandemic (*n* = 3). Lastly, some interviewees indicated that they relied on home remedies in an attempt to treat their illness (*n* = 3).

## Discussion

During the COVID-19 pandemic in the United States, xenophobic stigma and pandemic related nationalism further amplified the pre-existing underlying vulnerabilities of migrants; the pandemic brought into sharp relief the discrimination, racism, and social and health disparities migrants routinely encounter [25]. Migrants in high income countries also faced language barriers and encountered reduced health care coverage related to their immigration status– leading to higher risk of exposures, infections, and poor outcomes from COVID-19 [4]. The experiences of this study’s participants during the first year of the COVID-19 pandemic from 2020–2021 were consistent with these prior findings. A majority of asylum seekers reported experiences of oppression directly related to their migrant status, barriers to accessing health care during the pandemic, a lack of health insurance or primary care physician, and reliance on crowded emergency departments for treatment which subsequently raised concerns about exposure to COVID-19 and often deterred care seeking behavior.

The deleterious combination of decreased access to healthcare, increased risk of COVID-19 infection, and chronic mental health conditions has also been documented in the literature as a cause of high stress levels which exacerbate underlying mental health issues [26]. This study confirmed this observation and corroborated that the specific stressors of overcrowding, lack of health care, loneliness, financial strain, and disease-related stigma all affected the overall well-being of asylum seekers during the pandemic and increased their levels of psychological distress [7]. About half of the asylum seekers noted that their symptoms related to anxiety, depression, or posttraumatic stress disorder were exacerbated by the pandemic. Yet, COVID-19 also directly led to decreased access of mental health resources among migrants, resulting in unaddressed needs in this community [27]. In addition to mental health concerns, employed asylum seekers who were interviewed often faced discrimination, exploitation, and housing insecurity due to lack of money and lack of community support, as was documented in other immigrant families during the pandemic [28].

Although migrants in one prior study were found to have inadequate information during the pandemic [4], the knowledge and understanding about COVID-19 possessed by our study’s participants was highly accurate, with most of it obtained through television, the internet, and social media platforms. This was especially true regarding knowledge of social distancing, disease prevention through mask wearing, and hygiene precautions. Nonetheless, approximately half the asylum seekers experienced COVID-19 like symptoms and presumed COVID-19 infections, in large part due to their inability to adequately practice social distancing at home, in crowded living spaces, and on public transportation.

There have been multiple proposed strategies for providing services to asylum seekers, including promoting access to COVID-19 testing, directly addressing stress and mental health, focusing on individual’s social needs, and utilizing virtual care to improve access [29]. While these proposed paths will support asylum seekers currently living in the U.S., the Title 42 public health order enacted by the government early in the COVID-19 pandemic complicated matters. Title 42, which after a protracted court battle was discontinued,, put asylum seekers at increased risk of acquiring COVID-19 by refusing them admittance into the U.S., and by forcing them into crowded and inhumane conditions if they were able to enter the country. These Title 42 expulsions subjected asylum seekers, who were not the drivers of COVID-19 cases in the U.S. [30] to situations that were ripe for the virus’ transmission. Such U.S. immigration policies thus put asylum seekers at increased risk of new COVID-19 infections, further shaping this population’s pandemic experiences.

By bringing heightened awareness of governmental policies and discriminatory practices surrounding the pandemic and immigrant populations, we hope this may enlighten future public health measures in future pandemics and epidemics. This is crucial since, as this study demonstrated, in spite of their robust understanding about preventing virus symptoms and transmission, asylum seekers to the U.S. often faced multiple hardships during the first year of the COVID-19 pandemic such as difficulties accessing care, prejudice, employment exploitation, food and housing insecurities, and worsening overall health– all of which affect both exposure to COVID-19 and health outcomes. Future public health policies need to underline and target these drivers of illness and social determinants of health in order to address health disparities. Enhancing health networks for newly arrived immigrants, bolstering local healthcare frameworks, and allocating emergency benefits that will reach citizens and non-citizens alike will all be necessary to promote health equity in the face of inherent risks and challenges of future global pandemics.

## Limitations

All participants originated from one human rights program located in a medical institution in a large metropolitan region. As such, the generalizability of results to a wider community of asylum seekers during the COVID-19 pandemic and to those presenting to programs in different geographies is limited. In addition, all clients had secured legal representation at the time of evaluation, which may be reflective of the their migration experiences, social networks, health literacy, and health access upon arrival in the U.S. Future studies should investigate the experiences of a broader base of asylum seekers, including those who are seeking asylum as *pro se* litigants (without legal representation) to determine if differences exist in experiences during the COVID-19 pandemic, including accessing healthcare. Finally, this study addresses the experiences of asylum seekers during the first year of the COVID-19 pandemic. Follow up studies to understand this population’s health status during later waves of the pandemic will be important to undertake, specifically regarding their access to vaccines against the virus when they became available, as well as to oral antiviral agents to treat high risk patients and those with moderate to severe infections.

## Conclusion

Asylum seekers have been chronically underserved, and it appears that the pandemic only widened the gap in physical and mental health care. We sought to investigate the effect that the pandemic had on the refugee and asylee community through qualitative analysis, focusing on capturing the effects of the pandemic on their health, their access to health care, their health knowledge, disruptions to their daily lives, and their ability to take preventative measures against COVID-19. By exploring the experiences of asylum seekers in the U.S. during the first year of the COVID-19 pandemic, we have shed light on the socio-economic stressors affecting this population as well as their access to medical information, healthcare, and testing. This study contributes to the existing literature by highlighting the impact of the pandemic on this population’s health.

Our findings demonstrate that the social determinants of health greatly affected asylum seekers during the pandemic. Asylum seekers faced a range of discriminatory practices, particularly in employment opportunities, navigated underlying chronic physical and mental health issues, often lived in crowded spaces with difficulty quarantining, and encountered barriers to health care access during the pandemic—all of which impacted their well-being. We hope this study will spark dialogue about actions to improve asylum seekers’ health. Clinicians should work to provide comprehensive care and aim to address the social determinants of health of asylum seekers at all times, but particularly when strains on the healthcare system render asylum seekers’ access to care particularly precarious. Furthermore, the results of this study highlight the fact that asylum seekers possessed good health literacy, with a particular understanding of COVID-19 and knowledge of the public health measures needed to prevent its spread. This may inform future health education and harm reduction initiatives geared toward this population.

## Data Availability

The datasets (quotes from patient interviews) are included within the article. The corresponding author can be contacted to discuss Methods and data analysis.
